# Mental Health Factors Related to Quality of Life in Older Adults Using Long-Term Care Services in Mexico

**DOI:** 10.3390/healthcare13212769

**Published:** 2025-10-31

**Authors:** Christian Díaz de León-Castañeda, Ana Celia Anguiano-Morán, Elva Rosa Valtierra-Oba, Barbara Monica Lemus-Loeza, Gabriela Galván-Villalobos, Ericka Ivonne Cervantes-Pacheco, Christian Cortés-Rojo, Rocío Montoya-Pérez, Alaín Raimundo Rodríguez-Orozco

**Affiliations:** 1 Investigadoras e Investigadores por México, Secretaría de Ciencia, Humanidades, Tecnología e Innovación (SECIHTI), Ciudad de México 03940, Ciudad de México, Mexico; cddeleon@secihti.mx; 2 Facultad de Enfermería, Universidad Michoacana de San Nicolás de Hidalgo (UMSNH), Morelia 58260, Michoacán, Mexico; ana.anguiano@umich.mx (A.C.A.-M.); elva.valtierra@umich.mx (E.R.V.-O.); barbara.lemus@umich.mx (B.M.L.-L.); 3 Facultad de Ciencias Médicas y Biológicas “Dr. Ignacio Chávez”, Universidad Michoacana de San Nicolás de Hidalgo (UMSNH), Morelia 58020, Michoacán, Mexico; gabriela.galvan@umich.mx; 4 Facultad de Psicología, Universidad Michoacana de San Nicolás de Hidalgo (UMSNH), Morelia 58110, Michoacán, Mexico; ericka.cervantes@umich.mx; 5 Instituto de Investigaciones Químico-Biológicas, Universidad Michoacana de San Nicolás de Hidalgo (UMSNH), Morelia 58040, Michoacán, Mexico; christian.cortes@umich.mx (C.C.-R.); rocio.montoya@umich.mx (R.M.-P.)

**Keywords:** quality of life, older adults, depression, anxiety, cognitive reserve, mental health

## Abstract

**Background:** Older adults are a vulnerable population wherein their advancing age leads to limitations in physical and mental functionality that can compromise quality of life. **Objective:** The objective of this study was to analyze the relationship between mental health factors and quality of life in older adult users of long-term care services in Mexico. **Methods:** The present cross-sectional study was conducted with a convenience sample of 131 older adult users of long-term care services (three residential care homes and a day center) in Morelia, Michoacán, Mexico. A questionnaire including the World Health Organization Quality of Life Older Adults Scale (WHO-QoL-Old), Geriatric Depression Scale (GDS), Hamilton Anxiety Rating Scale (HARS), Cognitive Reserve Questionnaire (CRQ), and sociodemographic variables was administered. The analysis of the relationship between variables was performed using bivariate analysis (comparisons between groups and Pearson correlations). Due to the type of sampling, the representativeness of the sample obtained was not evaluated. **Results:** Depression and anxiety were found to inversely influence overall quality of life and its dimensions, while cognitive reserve is a factor that favors quality of life. Also, as related to cognitive reserve, level of education was found to be a factor that favors quality of life. **Conclusions:** Older adult users of long-term care services are a vulnerable group, given the negative impact on their quality of life that some mental health conditions could have, such as depression, anxiety, and low cognitive reserve.

## 1. Introduction

Mexico and various countries in the Latin American and Caribbean (LAC) region are undergoing demographic and epidemiological transitions, characterized by a higher proportion of older adults in the population who require more and better health services; therefore, promoting health and monitoring the living conditions of this age group is very important. Regarding the burden of disability through the *Years Lived with Disability* (YLD) for adults aged 70 and older living in Mexico, in 2021 a total rate of 28,217.5 YLD per 100 thousand was estimated, of which the ten principal causes were: diabetes (11.34%), age-related hearing loss (9.76%), blindness and vision loss (6.83%), low back pain (6.11%), osteoarthritis (5.38%), other musculoskeletal disorders (5.28%), Alzheimer’s disease and other dementias (4.87%), oral disorders (4.49%), falls (3.99%), and depressive disorders (2.72%) [1]. The high burden of these chronic diseases and mental health problems can compromise the quality of life of older adults across a variety of facets.

Quality of life has been defined in various ways by different sciences and disciplines, including political science, sociology, economics, psychology, and health sciences, with an evolution of this construct [2]. It has been recognized that quality of life is a construct that can be based on the perceptions of individuals in their social, socioeconomic, and cultural context. From the health sciences, quality of life has been defined by the World Health Organization (WHO) as “the perceptions of individuals regarding their position in life in the context of the culture and value systems in which they live and in relation to their goals, expectations, standards, and concerns” [3].

Additionally, in the health sciences field, for the assessment of quality of life three main instrumental approaches have been developed: a first approach, focused on assessing physical and mental functionality, principally known as *health-related quality of life* (HR-QoL); a second approach, focused on assessing aspects related to the *well-being* concept, such as autonomy, independency, dignity, comfort, social interaction, and personal projects accomplishment; and a third approach, that considers *well-being* is dependent on individuals’ functioning, so integrates HR-QoL dimensions [4]. Thus, the use of various conceptual frameworks and evaluation approaches for different purposes, both generic and specialized in specific conditions or age groups, has enabled the development of numerous instruments for evaluating the quality of life in the field of health.

Several dimensions or facets have been explicitly identified for assessing the quality of life in older adults [4,5,6]. For example, an important development in this field is the WHO–Quality of Life–Older adults Scale (WHO-QoL-Old) [7,8], that comprises six factors or facets focused on evaluating essential components of *well-being* in this age group, that in turn, depend of physical and mental functionality: (1) Sensory abilities (SAB) (defined as “Sensory functioning, impact of loss of sensory abilities on quality of life”); (2) Autonomy (AUT) (defined as “Independence in old age; being able or free to live autonomously and to take own decisions”); (3) Past, present, and future activities (PPF) (defined as “Satisfaction about achievements in life and at things to look forward to”); (4) Social participation (SOP) (defined as “Participation in activities of daily living, especially in the community”); (5) Death and dying (DAD) (defined as “Concerns, worries and fears about death and dying”); and (6) Intimacy (INT) (defined as “Being able to have personal and intimate relationships”) [8].

Evaluating and analyzing factors related to quality of life and *well-being* is particularly important for older adults living in vulnerable conditions and using long-term care services. The quality of life and *well-being* of these older adults can be influenced by several factors, such as sociodemographic factors (age, sex, educational attainment, marital status); socioeconomic factors (income, accessibility to health services); social factors (social support networks); *physical health* factors (presence of chronic non-communicable diseases [NCDs], health or risk behaviors); and *mental health* factors such as depression, anxiety, stress, or cognitive impairment [9,10]. In particular, studying mental health problems as a group of factors that could hinder the quality of life or *well-being* of older adults, there is an extensive body of published literature that has found important inverse associations between these problems and the quality of life or well-being [11,12,13,14,15,16,17]. However, this literature primarily originates from developed countries, within their social, socioeconomic, cultural, and health system contexts.

In the context of Mexico and other Latin American countries, there is scarce literature on evaluating the quality of life of older adults who use public or private long-term care services, analyzing the impact of related factors, including mental health factors. While a previous study conducted in Mexico and Ecuador evaluated the quality of life in older adults, it did not describe the specific outcomes for older adults using long-term care services, nor did it assess the impact of mental health conditions [18]. Another study assessed the impact of different physical and mental health factors on the autonomy dimension only [19].

The long-term care services are a broad group of services that can meet the different needs of older adults, offering interventions for healthcare needs, palliative care needs, or social care and support needs, that can be provided in different settings such as home, day care centers, community, and residential care homes. These services can be public or private and involve the participation de different caregivers and health professionals [20,21]. As part of long-term care services, day stay programs offer part-time daycare for older adults, focusing primarily on promoting social and recreational activities. In contrast, residential care homes (or nursing homes) provide care and attention throughout the day and for extended periods, often including medical care and the services of other health professionals. However, the package of services in both modalities may vary depending on the country’s health and social assistance systems, financing schemes, and users’ ability to pay [21,22].

In Mexico, public long-term care services for vulnerable or without payment capacity older adults are supported or implemented by the *Sistema para el Desarrollo Integral de la Familia* (DIF or System for Integral Family Development), a decentralized public agency funded by the Federal and State governments, and to a lesser extent by contributions from users. The DIF system has developed several programs to finance and support the operation de public or private day stays and residential care homes for older adults. The day stays supported by DIF consists of a part-time daycare for older adults, providing them with accommodation, food, medical, odontological, and psychological care, physiotherapy, social work, and recreational activities. In contrast, public residential care homes (supported by DIF) offer a broader package of services, including permanent residence, food, medical and psychological care, medication management, assistance with activities of daily living (ADLs), and recreational activities. Furthermore, there are private long-term care services for people with the ability to pay, with their respective service packages; although other private long-term care services operate through civil associations or religious organizations, focused on people without the ability to pay (receiving support from public welfare or religious organizations). The DIF has a support program for private residential care homes that attend vulnerable older adults, such as those in a situation of abandonment. It should be noted that these public or private long-term care services can benefit older adults with different types of health insurance, with or without Social Security.

The present study aims to analyze the mental health factors related to quality of life in older adult users of long-term care services in Morelia, Michoacán, Mexico.

## 2. Materials and Methods

### 2.1. Participants

A total of 131 older adults participated in the present study, which was conducted in 2023. The participants were users of public or private long-term care services, either residential care homes (*n* = 90) or a day stay center (*n* = 41), located in the city of Morelia, Michoacán, Mexico. Morelia is the capital of the Michoacán state and is the most important municipality of this state (743,275 inhabitants in 2020 [23]). Michoacán state is located in the central western region of Mexico (the 16th most populated state, with 4,748,846 inhabitants in 2020 [23]).

Three residential care homes and one day stay center located in the city of Morelia, Michoacán, were selected by convenience sampling. The residential care homes selected included different types of financing: one setting was exclusively private, financed only by contributions from users or relatives; one setting was private, although receiving support from the Church, public welfare, and small contributions from users; and one setting was private, but received support from DIF and public welfare. The day stay center is a public facility supported by DIF and the Municipal Government. All older adults using these centers during the study period (January 2024–February 2025) were considered for inclusion in the sample; only those without the ability to answer the questionnaires were excluded. Table 1 presents the main sociodemographic characteristics of the participants.

### 2.2. Instruments

The present study developed a structured questionnaire to explore the sociodemographic variables of interest and the following psychometric scales:

*World Health Organization—Quality of Life—Older adults Scale* (WHO-QoL-Old) [7,8]. This is a self-administered scale consisting of a total of 24 items distributed across the six factors or facets principally focused on evaluating *well-being*: (1) Sensory abilities (SAB) (defined as “Sensory functioning, impact of loss of sensory abilities on quality of life”); (2) Autonomy (AUT) (defined as “Independence in old age; being able or free to live autonomously and to take own decisions”); (3) Past, present, and future activities (PPF) (defined as “Satisfaction about achievements in life and at things to look forward to”); (4) Social participation (SOP) (defined as “Participation in activities of daily living, especially in the community”); (5) Death and dying (DAD) (defined as “Concerns, worries and fears about death and dying”); and (6) Intimacy (INT) (defined as “Being able to have personal and intimate relationships”) (WHO-QoL-Old) [8]. Each factor consists of four items, and each item has five response categories, although the nomenclature for these categories may vary depending on the item and factor. Total scores range from 24 to 120. It is worth highlighting the relevance of this scale in evaluating the quality of life of older adults in the well-being domain, as it has sufficient evidence of validity and reliability, which have been tested in different languages and countries. The present study utilized the Spanish version, as adapted in Mexico, to incorporate iconography that graphically represents the categories of responses received for each item [24,25]. A previous study analyzed the validity of this scale based on its internal structure and relationship with other variables, as well as its internal consistency [26]. The coefficients alpha obtained by the present study for the six factors were found to be good (SAB: 0.818, AUT: 0.781, PPF: 0.808, SOP: 0.858, DAD: 0.853, and INT: 0.913).

*Geriatric Depression Scale* (GDS) [27]. This is a self-administered scale consisting of 30 items that could be considered unidimensional, although some studies have identified diverse factors within it [28]. Each item is dichotomized into yes or no responses. The score is calculated by counting the responses that suggest depression (reversing the inverse items). Total scores range from 0 to 30. The cut-off points for the evaluation of depressive disorders are 0–9, “Normal”; 10–19, “Mild”; and >20, “Severe”. The present study used the Spanish version of the 30-item GDS scale, which was translated in Spain [29] and has been subsequently adapted for use in Mexico [30]. The coefficient alpha obtained by the present study for the 30-item unidimensional scale was found to be good (α = 0.916).

*Hamilton Anxiety Rating Scale* (HARS) [31]. This is a clinician-rated instrument comprising 14 items and encompassing two factors: (1) somatic anxiety and (2) psychic anxiety. Each item is rated via five response levels of intensity, ranging from 0 (not present) to 4 (very severe). The total possible scores range from 0 to 56. The cut-off points for the evaluation of anxiety are as follows: 0–17, “Mild”; 18–24, “Moderate”; and >27, “Severe”. The present study used a Spanish version of the HARS scale [32], which has been adapted and recommended for use by clinical practice guidelines corresponding to public health services in Mexico [33]. The coefficients alpha obtained by the present study for the somatic and psychic factors were found to be good (Somatic dimension: 0.777, Psychic dimension: 0.851).

*Cognitive Reserve Questionnaire* (CRQ) [34]. This is a self-administered questionnaire, originally developed in Spanish. It consists of eight items, each revealing a cognitive reserve factor: level of education, parents’ level of education, training courses, occupation throughout one’s life, musical training, languages spoken, reading level, and playing intellectual games. There are between three and six answer options (scoring from 0 to 6) for these items. The total score of the questionnaire ranges from 0 to 25. The developers of the CRQ questionnaire proposed three cut-offs based on quartiles used for the evaluation of cognitive reserve, as follows: 0–6, “Inferior range”; 7–9, “Medium-low range”; 10–14, “Medium-high range”; and >15, “Upper range”. Some studies have contributed to the validity and reliability evidences of this questionnaire in older adults [35,36].

Questionnaire-printed forms were interviewer-administered to all the participants. Interviewers were nursing professionals and co-authors of this study.

### 2.3. Data Analysis

#### 2.3.1. Descriptive Analysis 

The present study performed a descriptive analysis on both the sociodemographic variables identified in the participants and the scores obtained for the psychometric scales. The inferential normality of the scores obtained for the WHO-QoL-Old scale and its dimensions (as dependent variables) was evaluated using the Kolmogorov–Smirnov test to determine which statistical tests to use in the subsequent analyses.

Figure S1 (Supplementary Material) shows a conceptual model used to guide the statistical analysis.

#### 2.3.2. Bivariate Analysis

Groups comparison analyses were performed to compare the total scores obtained using the WHO-QoL-Old scale and its dimensions by category, such as the sociodemographic variables (sex, age, or level of education), and the levels of the variables of interest identified using the psychometric scales of interest (GDS, HARS, and CRQ). A Student’s *t*-statistical test (Mann–Whitney’s U test, for data not complying with normality) was applied for two groups, while the ANOVA test (Kruskal–Wallis test, for data not complying with normality) was used for three or more groups. Effect size measures, such as Cohen’s d and squared eta (η^2^), were computed for two and three (or more) groups comparisons, respectively [37].

Pearson’s correlation analyses were performed between the total scores obtained using the WHO-QoL-Old scale and its dimensions and either the sociodemographic variables (age or years of education) or the scores obtained for the psychometric scales of interest (GDS, HARS, and CRQ).

The inferential hypotheses were tested with a significance level (α) of 0.050. Statistical power was computed for both bivariate analyses (group comparisons and Pearson’s correlations).

#### 2.3.3. Software

The SPSS 25 program was used for the management and statistical analysis of the information collected. G*Power software (ver. 3.1.9.7) was used to compute statistical power.

### 2.4. Ethical Considerations

The research protocol was approved by the Committee of the Morelia Institute for Research in Pharmacology, Allergy, and Immunology (Approval number: P0224).

## 3. Results

### 3.1. Descriptive Analysis of the Sociodemographic Variables and Psychometric Scales

Table 1 presents the descriptive analysis of the sociodemographic characteristics of the participants and the scores obtained using the psychometric scales. Most participants were residential care home users (68.7%), while the remainder were day stay service users (31.3%). The mean level of education was ten years (SD = 5.0 years), with a high percentage of participants having primary schooling or less (35.9%). The descriptive analysis conducted on the results obtained using the mental health scales showed high frequencies of moderate or severe depression (80.9%), moderate or severe anxiety (49.6%), and cognitive reserve in the lower range (38.2%).

### 3.2. Group Comparison Analysis

Table 2 shows the group comparisons undertaken for the total scores obtained using the WHO-QoL-Old scale and its dimensions, by sociodemographic variables. No statistically significant differences in quality of life and its dimensions were found between the sex groups (d ranged from 0.00 to 0.28). For age, a similar result was found, with no statistically significant differences in quality of life and its dimensions between the age groups, except for the SAB dimension (d = 0.43). Also, it was found that a higher level of education corresponds to a higher quality of life, with statistically significant differences found among education groups for the WHO-QoL-Old scale and its dimensions, except for the DAD dimension; a higher effect size (η^2^) was obtained for the AUT dimension (η^2^ = 0.30).

Comparing the effect sizes of these sociodemographic factors on the WHO-QoL-Old scale total scores, the education level showed the highest effect (η^2^ = 0.21), while sex and age had similar effect sizes (η^2^ = 0.18 and 0.13, respectively).

Table 3 shows the group comparisons undertaken for the total scores obtained using the WHO-QoL-Old scale and its dimensions, by mental health factors. A higher level of depression was found to be related to a lower level of quality of life, while statistically significant differences among groups were obtained for the WHO-QoL-Old scale and its dimensions, except for the DAD and INT dimensions; the highest effect size was obtained for the AUT dimension (η^2^ = 0.18). A similar result was obtained for anxiety, although a significant association with the DAD dimension was also obtained; the highest effect size was obtained for the SOP dimension (η^2^ = 0.19). It was found that a higher cognitive reserve is related to a better quality of life, while statistically significant differences among groups were also obtained with the WHO-QoL-Old scale and its dimensions, except for the DAD dimension; the highest effect size was obtained for the AUT dimension (η^2^ = 0.32).

Comparing the effect sizes of these three mental health factors on the WHO-QoL-Old scale total scores, the cognitive reserve showed the highest effect (η^2^ = 0.27), while depression and anxiety had similar effect sizes (η^2^ = 0.15 and 0.17, respectively).

### 3.3. Pearson Correlation Analysis

Table 4 shows the Pearson correlations for the factors of interest (on a numerical scale) and the WHO-QoL-Old scale and its dimensions. Similar results to those identified by the group comparisons were found, both in inferential hypothesis tests and effect sizes. No statistically significant correlations were found for age. Statistically significant correlations were obtained for the following: years of education, in the range of 0.282 to 0.496 (highest correlation with AUT), and no significant correlation with the DAD dimension; depression, in the range of −0.222 to −0.396 (similar correlations with AUT, PPF, and SOP), and no significant correlation with the DAD and INT dimensions; anxiety, in the range of −0.193 to −0.493 (highest correlations with PPF and SOP); cognitive reserve, in the range of 0.324 to 0.510 (highest correlation with AUT), and no significant correlation with the DAD dimension.

## 4. Discussion

The present study revealed several important findings regarding factors related to the quality of life of older adult users of long-term care services in Mexico.

### 4.1. Relationship with Sociodemographic Variables

No difference in quality of life was found between the sexes. While other studies using the same scale found variable results, the results obtained by some have coincided with the results of the evaluation conducted with the global scale [12,38], and others have found a lower quality of life in women [39].

A significant difference was only found for age in the SAB dimension, for which those under 75 years of age presented a better score, an expected finding given that older age leads to both a deterioration in sensory skills and wear and tear on physical function in general. Other studies that have used the same scale have also identified a deterioration, in line with age, in quality of life for this dimension and other dimensions, such as AUT and DAD [40]. This association has also been reported for the total scale [12].

For most dimensions of quality of life, a higher level of education was found to correlate with a better quality of life, a finding observed in both the group comparisons and the correlation analysis. Only for the DAD dimension was this association not found in the group comparisons or correlation analysis. Similarly, other studies have found statistically significant relationships between level of education and overall quality of life assessed on the same scale and its various dimensions [38,40]. In this regard, it should be noted that educational level can also be correlated with socioeconomic level, with other studies having also found a significant relationship with this variable.

### 4.2. Relationship with Depression

The group comparison and Pearson’s correlation analysis obtained the same results for depression. The bivariate analysis identified that having depression is related to a lower quality of life both in the general scale and in the SAB, AUT, PPF, and SOP dimensions. This association with depression could be related to reduced physical activity impacting the SAB dimension, feelings of dependence on other people affecting the AUT dimension, feelings of failure and hopelessness affecting the PPF dimension, and a sense of apathy affecting the SOP dimension. However, no statistically significant relationship was found with the DAD and INT dimensions in either of the two bivariate analyses. In relation to the DAD dimension, it is worth mentioning that depression itself can be related to a wide range of symptoms such as sadness, hopelessness and even desires to harm or die. In relation to the INT dimension, it is worth mentioning that the older adults who made up the sample were mostly residents in residential care homes, where it is difficult to have a sex life, in fact, this dimension was the one that had the lowest overall score compared to the other dimensions.

Several published studies have also found an inverse relationship between depression and overall quality of life, as assessed using the same scale as the one used in the present study [11,12,13]. In particular, a previous study found a similar pattern of results to those found by the present study, characterized by a significant relationship between depression and all dimensions except for DAD and INT, although no relationship with the global score was identified [14].

### 4.3. Relationship with Anxiety

The group comparison and Pearson’s correlation analysis obtained similar results. Presenting anxiety was found to be related to lower quality of life both in the general scale and in the SAB, AUT, PPF, SOP, and DAD dimensions. Similarly to the findings found for depression, anxiety could be related to the following: reduced physical activity affecting the SAB dimension; feelings of uselessness or dependence on caregivers or other people affecting the AUT dimension; feelings of hopelessness or anguish towards present and future activities affecting the PPF dimension; the need for isolation or the avoidance of social circles affecting the SOP dimension; or fear of the future affecting the DAD dimension. With the INT dimension, a relationship not statistically significant was found through the group comparison analysis; however, the correlation analysis was significant but had the highest *p* value. Similarly to the results obtained for the relationship between this dimension and depression, these findings can be attributed to the sample composition, which is primarily composed of the elderly living in residential care homes.

Some published studies have also found an inverse relationship between anxiety and the overall quality of life assessed using the same scale as the present study [11]. Additionally, another study also found an inverse relationship with the AUT dimension [19].

### 4.4. Relationship with Cognitive Reserve

The group comparisons and Pearson’s correlation analyses conducted in the present study yielded the same results for cognitive reserve. A higher cognitive reserve was found to be associated with a higher quality of life, as measured by both the general scale and the SAB, AUT, PPF, SOP, and INT dimensions. Low cognitive reserve could be related to problems in quality of life in different ways, like the following: a lower capacity to perform physical activity affecting the SAB dimension; a feeling of reduced ability to perform activities autonomously affecting the AUT dimension; a perception of a lower capacity to carry out personal activities and projects affecting the PPF dimension; a perception of a lower capacity to perform social activities affecting the SOP dimension; or a perception of a lower capacity to live a healthy life as a couple affecting the INT dimension.

For the DAD dimension, no statistically significant relationship was found in either of the two bivariate analyses. This result could be explained by the fact that, regardless of the educational level and cognitive reserve of the elderly, perceptions about death and agony may exist.

Several other published studies have also found a direct relationship between cognitive reserve and overall quality of life, as assessed using the same scale as the present study [26] or specific dimensions, such as AUT [19]. In particular, one study found the same pattern of statistically significant relationships as those observed in the present study, revealing a significant relationship between cognitive reserve and the global quality of life scale, as well as all its dimensions, except DAD [14]. Only one study has reported no statistically significant relationship between cognitive reserve and quality of life, particularly in its SOP dimension [15].

### 4.5. Final Reflections Regarding the Results Obtained in the Study

In general terms, it is worth mentioning that given the complexity of the scale used to assess quality of life (from a closer approach to *well-being*), the dimensions or facets of the WHO-QoL-OLD scale are very different from each other, so different results could be expected in the analysis of its relationship with sociodemographic and mental health factors, as were shown in this study through statistical significance or *p* values. Additionally, the differing results presented in this study compared to those found in other countries may be attributed to the different social and cultural contexts. It is worth noting that the social and cultural context can have a significant impact on various dimensions of quality of life, particularly from the perspective of *well-being*.

### 4.6. Strengths and Weaknesses of the Study

The use of the quality-of-life scale is identified as a strength of the present study, as it integrates several dimensions or facets of the quality of life of older adults, which has evidence of validity and reliability. Moreover, valid and reliable scales were used for evaluating aspects related to mental health in the fields of depression, anxiety, and cognitive reserve.

A cross-sectional study design is limited in its ability to infer causal relationships and can be mentioned as a weakness. Likewise, the sample design, based on convenience sampling of long-term care services (residential care homes and day care centers), could imply a possible selection bias, or may have obtained a sample that does not accurately represent the population proportions of older adults who use the different types of services. Another limitation identified is the exclusion of people who were unable to complete the questionnaires, despite the support of the interviewers. All these aspects could compromise the representativeness of the sample obtained.

Additionally, considering the total number of older adult users of long-term care services in Michoacán or the entire country, the sample size could be regarded as a weakness. Additionally, this limitation hindered the statistical analyses, which were performed using bivariate analysis only. This approach may have some implications, as the relationship between mental health factors and quality of life is not controlled for other variables that may be involved, such as education, income, and type of care setting.

In this sense, it is proposed that subsequent studies aim to obtain a larger sample size using complex probabilistic sampling, considering the different kinds of long-term care services and the entire older adult population that uses them in the country, in order to achieve better representativeness and external validity. A larger sample size will allow multivariate analysis, such as multiple linear regression analysis, with greater statistical power. Another proposal for future research in this area is the evaluation of the impact of other variables on quality of life and its dimensions, such as physical health as measured through functional status indicators (i.e., Activities of Daily Living [ADL] and Instrumental Activities of Daily Living [IADL]) and the presence of chronic non-communicable diseases and healthy or risk behaviors, as well as the evaluation of socioeconomic level through income. Additionally, the use of longitudinal tracking studies would be recommended to understand the mechanisms by which each factor influences the quality of life dimensions. Additionally, this study could be extended to explore this analysis in older adults who are not institutionalized (open population).

### 4.7. Clinical Implications

As implications for health professionals, the importance of the evaluation of mental health conditions and quality of life in older adults who use long-term care services is identified. The use of the psychometric scales used in this study may be recommended as an additional support to the clinical evaluation.

### 4.8. Implications for Public Health and Policy

Through the results of this study, several recommendations for public health and policy in Mexico can be identified, including the development or strengthening of mental health programs to ensure coverage of mental healthcare services for the elderly who utilize public and private long-term care services, such as residential care homes and day stays, as well as the general (non-institutionalized) population. To achieve this in the public sector, it could be feasible to create programs that promote the strengthening of mental healthcare provision through the improvement of human resource availability (psychologists and psychiatrists) at the primary care level of healthcare institutions (i.e., *Servicios Estatales de Salud* [State health services] aimed at the population without formal employment, or the main social security institutions, *Instituto Mexicano del Seguro Social* [IMSS] and the *Instituto de Seguridad y Servicios Sociales de los Trabajadores del Estado* [ISSSTE]), also, special programs to perform mental healthcare in community settings, residential care homes, day stays should be implemented. Another strategy could be to promote the recruitment and retention of mental health personnel in the public long-term care services. Additionally, the *Secretaría de Salud* (Ministry of Health) should strengthen its stewardship to promote the provision of mental health services in private long-term care facilities.

The presence of mental health professionals could have numerous benefits for older adults through the implementation of strategies such as promoting cognitive stimulation, or prevention and treatment of depression or anxiety. On a broader scale, it is essential to enhance mental health policy and governance in Mexico.

## 5. Conclusions

This study examined the relationship between mental health and sociodemographic factors and the quality of life of older adult users of long-term care services in Mexico. Among these older adults, multiple facets of their quality of life or *well-being* were found to be related to mental health conditions, such as anxiety, depression, and cognitive reserve. Additionally, certain sociodemographic characteristics were found to be important for quality of life, such as advanced age and level of education. These findings show the importance of strengthening public mental health programs aimed at older adults in long-term care services.

## Figures and Tables

**Table 1:** Sociodemographic and clinical characteristics of participants (*n* = 131).

Variable	Category	*n*	%
Sex (*n*, %)	Women	91	69.5
	Men	40	30.5
Age, in years, (M, SD)		M = 77.3	SD = 7.9
Age group (*n*, %)	≤75 years	57	43.5
	>76 years	74	56.5
Public program use (*n*, %)	Residential care home	90	68.7
	Day stay	41	31.3
Education, in years (M, SD)		M = 10.0	SD = 5.0
Education (*n*, %)	Illiterate	11	8.4
	Primary	36	27.5
	Secondary	25	19.1
	High school/baccalaureate or higher	59	45.0
Marital status (*n*, %)	Single	32	24.4
	Married	46	35.1
	Divorced	9	6.9
	Widow/widower	44	33.6
GDS (M, SD)		M = 17.0	SD = 7.9
Depression level (*n*, %) ^a^	Normal (<9)	25	19.1
	Moderate (10–19)	55	42.0
	Severe (>20)	51	38.9
HARS (M, SD)		M = 17.1	SD = 9.3
Anxiety level (*n*, %) ^b^	Mild (<17)	66	50.4
	Moderate (18–24)	44	33.6
	Severe (>25)	21	16.0
CRQ (M, SD)		M = 8.4	SD = 4.8
Cognitive reserve level (*n*, %) ^c^	Lower range	50	38.2
	Medium-low range	29	22.1
	Medium-high range	36	27.5
	Upper range	16	12.2

Acronyms: GDS: Geriatric Depression Scale; HARS: Hamilton Anxiety Rating Scale; CRQ: Cognitive Reserve Questionnaire.

^a^ Categories of depression, based on the cut-offs stipulated by the GDS Scores: 0–9, “Normal”; 10–19, “Moderate”; and >20, “Severe”.

^b^ Categories of anxiety, based on the cut-offs stipulated by the HARS Scores: 0–17, “Mild”; 18–24, “Moderate”; and >27, “Severe”.

^c^ Categories of cognitive reserve, based on the cut-offs stipulated by the CRQ Scores: 0–6, “Inferior range”; 7–9, “Medium-low range”; 10–14, “Medium-high range”; and >15, “Upper range”.

**Table 2:** Scores of the WHO-QoL-Old scale and its dimensions by groups of the sociodemographic factors (*n* = 131) ^a,b^.

		WHO-QoL-Old		SAB		AUT		PPF		SOP		DAD		INT	
	*n*	M (SD)	d/η^2^ *p* [Power]	M (SD)	d/η^2^ *p* [Power]	M (SD)	d/η^2^ *p* [Power]	M (SD)	d/η^2^ *p* [Power]	M (SD)	d/η^2^ *p* [Power]	M (SD)	d/η^2^ *p* [Power]	M (SD)	d/η^2^ *p* [Power]
**Total** **sample**	131	83.8 (16.6)	-	14.7 (3.4)	-	13.5 (3.6)	-	14.4 (3.5)	-	13.7 (3.9)	-	15.3 (4.1)	-	12.2 (4.2)	-
**Sex**															
Women	91	84.7 (16.4)	d = 0.18 0.340 [0.24]	14.9 (3.4)	d = 0.28 0.147 [0.42]	13.6 (3.6)	d = 0.07 0.723 [0.10]	14.6 (3.5)	d = 0.21 0.260 [0.30]	13.9 (3.9)	d = 0.11 0.558 [0.14]	15.3 (4.1)	d = 0.00 0.986 [0.05]	12.4 (4.1)	d = 0.15 0.422 [0.20]
Men	40	81.7 (17.0)		14.0 (3.5)		13.4 (3.5)		13.9 (3.3)		13.4 (3.9)		15.2 (4.3)		11.8 (4.5)	
**Age**															
≤75 years	57	85.0 (15.9)	d = 0.13 0.474 [0.16]	15.5 (3.0)	d = 0.43 **0.016** [0.73]	14.0 (3.4)	d = 0.23 0.196 [0.33]	14.3 (3.5)	d = −0.04 0.805 [0.08]	13.6 (4.0)	d = −0.03 0.844 [0.07]	14.9 (4.5)	d = 0.17 0.331 [0.23]	12.7 (3.9)	d = 0.20 0.255 [0.28]
>76 years	74	82.9 (17.2)		14.0 (3.6)		13.2 (3.6)		14.4 (3.5)		13.8 (3.8)		15.6 (3.8)		11.9 (4.4)	
**Education**															
Illiterate	11	64.3 (12.0)	**η^2^ = 0.21** **<0.001** [0.99]	11.4 (4.8)	**η^2^ = 0.15** **<0.001** [0.98]	8.5 (3.2)	**η^2^ = 0.30** **<0.001** [0.99]	9.9 (2.9)	**η^2^ = 0.18** **<0.001** [0.99]	9.7 (2.3)	**η^2^ = 0.14** **<0.001** [0.97]	16.5 (4.3)	η^2^ = 0.02 0.378 [0.28]	8.4 (3.7)	**η^2^ = 0.14** **<0.001** [0.99]
Primary	36	78.1 (14.5)		13.6 (3.3)		12.1 (3.1)		13.9 (3.1)		13.0 (3.3)		14.3 (3.8)		11.1 (3.8)	
Secondary	25	90.4 (15.4)		15.9 (2.2)		14.6 (3.4)		15.1 (3.2)		15.1 (3.9)		15.7 (3.4)		14.0 (4.4)	
Baccalaureate or higher	59	88.1 (15.4)		15.4 (3.1)		14.9 (2.7)		15.1 (3.3)		14.3 (3.9)		15.4 (4.5)		12.9 (3.8)	

Acronyms: SAB: Sensory abilities; AUT: Autonomy; PPF: Past, present, and future activities; SOP: Social participation; DAD: Death and Dying; INT: Intimacy.

^a^ Effect size measures: d = Cohen’s d; η^2^ = Squared eta. Categories of d values: <0.19, “Negligible”; 0.20–0.49, “Small”; 0.50–0.79, “Medium”; and >0.80, “Large”. Categories of η^2^ values: <0.01, “Negligible”; 0.01–0.06, “Small”; 0.06–0.14, “Medium”; and >0.14, “Large”. Bold show d or η^2^ “Large” values.

^b^ *p*-values obtained for the t-Student test (2-group comparisons) or the ANOVA test (three or more group comparisons). Non-parametric tests were used only for the DAD dimension (lack of normality test): Mann–Whitney’s U (2 groups comparisons) or Kruskal–Wallis test (three or more groups comparisons). Bold *p*-values show those that were statistically significant (*p* < 0.050).

**Table 3:** Scores of the WHO-QoL-Old scale and its dimensions by groups of the mental health factors (*n* = 131) ^d,e^.

		WHO-QoL-Old		SAB		AUT		PPF		SOP		DAD		INT	
	n	M (SD)	η^2^ *p* [Power]	M (SD)	η^2^ *p* [Power]	M (SD)	η^2^ *p* [Power]	M (SD)	η^2^ *p* [Power]	M (SD)	η^2^ *p* [Power]	M (SD)	η^2^ *p* [Power]	M (SD)	η^2^ *p* [Power]
**Total** **sample**	131	83.8 (16.6)	-	14.7 (3.4)	-	13.5 (3.6)	-	14.4 (3.5)	-	13.7 (3.9)	-	15.3 (4.1)	-	12.2 (4.2)	-
**Depression ^a^**															
Normal	25	95.6 (11.1)	**η^2^ = 0.15** **<0.001** [0.99]	15.9 (2.3)	η^2^ = 0.06 **0.021** [0.71]	16.0 (3.0)	**η^2^ = 0.18** **<0.001** [0.99]	16.7 (2.2)	η^2^ = 0.12 **<0.001** [0.97]	16.6 (2.9)	**η^2^ = 0.16** **<0.001** [0.99]	16.5 (4.0)	η^2^ = 0.03 0.189 [0.36]	13.9 (3.0)	η^2^ = 0.04 0.073 [0.53]
Moderate	55	84.2 (14.4)		15.0 (3.2)		13.9 (3.3)		14.3 (3.1)		13.8 (3.6)		15.2 (3.9)		12.0 (4.1)	
Severe	51	77.5 (18.0)		13.7 (3.9)		11.9 (3.3)		13.4 (3.8)		12.2 (3.8)		14.7 (4.3)		11.6 (4.6)	
**Anxiety ^b^**															
Mild	66	89.9 (14.5)	**η^2^ = 0.17** **<0.001** [0.99]	15.2 (3.1)	η^2^ = 0.08 **0.004** [0.87]	14.4 (3.8)	η^2^ = 0.07 **0.012** [0.78]	15.7 (3.2)	**η^2^ = 0.16** **<0.001** [0.99]	15.3 (3.4)	**η^2^ = 0.19** **<0.001** [0.99]	16.3 (3.6)	**η^2^ = 0.15** **<0.001** [0.99]	13.0 (4.3)	η^2^ = 0.04 0.090 [0.49]
Moderate	44	80.5 (15.8)		14.8 (3.3)		12.9 (3.2)		13.3 (3.2)		12.8 (3.8)		15.4 (4.1)		11.3 (3.6)	
Severe	21	71.5 (16.1)		12.4 (4.0)		12.2 (2.7)		12.3 (3.1)		10.9 (3.2)		11.8 (4.0)		11.9 (4.5)	
**Cognitive reserve ^c^**															
Lower range	50	73.3 (16.2)	**η^2^ = 0.27** **<0.001** [0.99]	12.9 (3.8)	**η^2^ = 0.19** **<0.001** [0.99]	11.0 (3.4)	**η^2^ = 0.32** **<0.001** [0.99]	12.5 (3.5)	**η^2^ = 0.18** **<0.001** [0.99]	11.7 (3.8)	**η^2^ = 0.18** **<0.001** [0.99]	15.0 (3.9)	η^2^ = 0.02 0.490 [0.23]	10.2 (4.5)	**η^2^ = 0.16** **<0.001** [0.99]
Medium-low	29	93.6 (11.6)		16.4 (2.3)		15.6 (2.4)		15.7 (3.0)		15.8 (2.8)		16.3 (3.9)		13.9 (3.5)	
Medium-high	36	87.2 (14.5)		15.1 (3.0)		14.6 (2.8)		15.4 (2.9)		14.2 (3.7)		14.8 (4.3)		13.1 (3.5)	
Upper range	16	91.3 (12.2)		16.1 (2.1)		15.3 (2.8)		15.6 (2.8)		15.1 (3.4)		15.3 (4.8)		13.9 (2.9)	

Acronyms: SAB: Sensory abilities; AUT: Autonomy; PPF: Past, present, and future activities; SOP: Social participation; DAD: Death and Dying; INT: Intimacy.

^a^ Categories of depression, based on the cut-offs stipulated by the GDS Scores: 0–9, “Normal”; 10–19, “Moderate”; and >20, “Severe”.

^b^ Categories of anxiety, based on the cut-offs stipulated by the HARS Scores: 0–17, “Mild”; 18–24, “Moderate”; and >25, “Severe”.

^c^ Categories of cognitive reserve, based on the cut-offs stipulated by the CRQ Scores: 0–6, “Inferior range”; 7–9, “Medium-low range”; 10–14, “Medium-high range”; and >15, “Upper range”.

^d^ Effect size measure: η**^2^** = Squared eta. Categories of η^2^ values: <0.01, “Negligible”; 0.01–0.06, “Small”; 0.06–0.14, “Medium”; and >0.14, “Large”. Bold show η^2^ “Large” values.

^e^ *p*-values obtained for the ANOVA test. The Kruskal–Wallis test was used only for the DAD dimension (lack of normality test). Bold *p*-values show those that were statistically significant (*p* < 0.050).

**Table 4:** Pearson’s correlations between factors of interest and the WHO-QoL-Old scale and its dimensions (*n* = 131) ^a,b^.

	WHO-QoL-Old	SAB	AUT	PPF	SOP	DAD	INT
	r (*p*) [Power]	r (*p*) [Power]	r (*p*) [Power]	r (*p*) [Power]	r (*p*) [Power]	r (*p*) [Power]	r (*p*) [Power]
**Age (years)**	−0.030 (0.737) [0.06]	−0.164 (0.061) [0.47]	−0.110 (0.213) [0.24]	0.064 (0.468) [0.11]	0.033 (0.706) [0.07]	0.087 (0.323) [0.17]	−0.059 (0.504) [0.10]
**Education (years)**	**0.382 (<0.001)** [0.99]	0.299 **(0.001)** [0.94]	**0.496 (<0.001)** [0.99]	**0.364 (<0.001)** [0.99]	0.284 **(0.001)** [0.92]	0.002 (0.982) [0.05]	0.282 **(0.001)** [0.92]
**Depression** **(GDS Score)**	**−0.359 (<0.001)** [0.99]	−0.222 **(0.011)** [0.73]	**−0.394 (<0.001)** [0.99]	**−0.344 (<0.001)** [0.99]	**−0.396 (<0.001)** [0.99]	−0.112 (0.205) [0.25]	−0.148 (0.091) [0.40]
**Anxiety** **(HARS Score)**	**−0.471 (<0.001)** [0.99]	**−0.304 (<0.001)** [0.95]	−0.298 **(0.001)** [0.94]	**−0.425 (<0.001)** [0.99]	**−0.493 (<0.001)** [0.99]	**−0.371 (<0.001)** [0.99]	−0.193 **(0.027)** [0.61]
**Cognitive reserve** **(CRQ Score)**	**0.432 (<0.001)** [0.99]	**0.402 (<0.001)** [0.99]	**0.510 (<0.001)** [0.99]	**0.389 (<0.001)** [0.99]	**0.324 (<0.001)** [0.97]	−0.013 (0.880) [0.05]	**0.341 (<0.001)** [0.98]

Acronyms: SAB: Sensory abilities; AUT: Autonomy; PPF: Past, present, and future activities; SOP: Social participation; DAD: Death and Dying; INT: Intimacy.

^a^ Effect size measure: Pearson’s r. Categories of Pearson’s r values: 0–0.10, “Negligible”; 0.10–0.29, “Small”; 0.30–0.49, “Medium”; 0.50–1.0, “Large”. Bold r values show “Medium” or “Large” results.

^b^ *p*-values obtained for the Pearson’s r test. Bold *p*-values show those that were statistically significant (*p* < 0.050).

## Data Availability

Data can be requested from the corresponding author via email.

## Supplementary Materials

The following supporting information can be downloaded at: https://www.mdpi.com/article/10.3390/healthcare13212769/s1, Figure S1: Conceptual model that relates mental health factors to quality of life (*well-being* approach) and its six dimensions.

## Abbreviations

The following abbreviations are used in this manuscript:

WHO-QoL-Old	World Health Organization—Quality of Life—Older adults Scale
SAB	Sensory abilities
AUT	Autonomy
PPF	Past, present, and future activities
SOP	Social participation
DAD	Death and dying
INT	Intimacy
GDS	Geriatric Depression Scale
HARS	Hamilton Anxiety Rating Scale
CRQ	Cognitive Reserve Questionnaire
