# Supplementary material for: Mental Health Factors Related to Quality of Life in Older Adults Using Long-Term Care Services in Mexico
Source: Healthcare (Basel). 2025 Oct 31;13(21):2769. doi: 10.3390/healthcare13212769 (PMC12608625; doi:10.3390/healthcare13212769)
Supplement: Supplementary file 1 [file healthcare-13-02769-s001.zip › healthcare-3872793-supplementary.pdf]

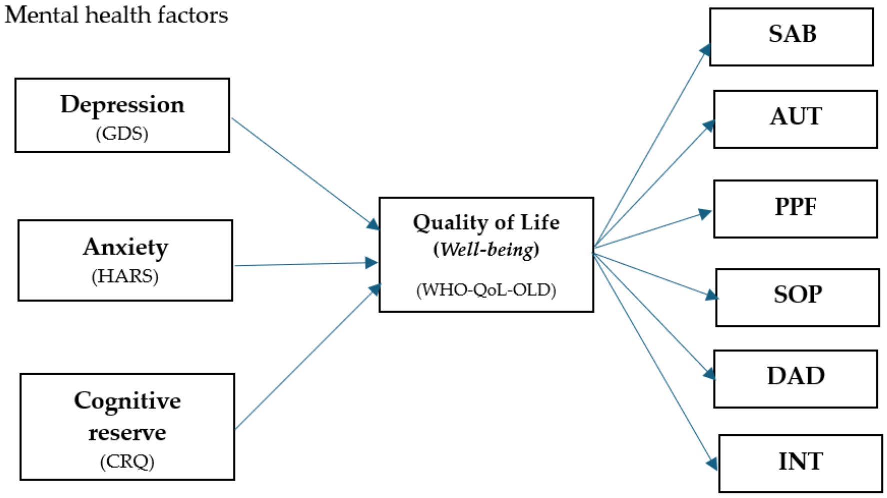

Figure S1. Conceptual model that relates mental health factors to quality of life (well-being approach) and its six dimensions

Acronyms: Quality of Life dimensions: SAB: Sensory abilities; AUT: Autonomy; PPF: Past, present, and future activities; SOP: Social participation; DAD: Death and Dying; INT: Intimacy.

Psychometric scales: WHO-QoL-Old: World Health Organization Quality of Life Older Adults Scale; GDS: Geriatric Depression Scale; HARS: Hamilton Anxiety Rating Scale; CRQ: Cognitive Reserve Questionnaire.
